# Effectiveness of Photobiomodulation With Low-Level Laser Therapy on the Implant Stability Quotient at Different Time Intervals: A Randomized Clinical Trial

**DOI:** 10.7759/cureus.52160

**Published:** 2024-01-12

**Authors:** Amrutha Shenoy, Dhanraj Ganapathy, Subhabrata Maiti

**Affiliations:** 1 Department of Prosthodontics and Implantology, Saveetha Dental College and Hospitals, Saveetha Institute of Medical and Technical Sciences, Saveetha University, Chennai, IND

**Keywords:** low-level laser therapy, osseointegration, implant stability quotient, dental implant surgery, photobiomodulation therapy

## Abstract

Background

Photobiomodulation techniques, particularly low-level laser therapy (LLLT), have gained traction due to their ability to accelerate osseointegration by stimulating cellular metabolism and promoting tissue healing. This study explores the effectiveness of LLLT around dental implants at various intervals after placement. Using resonance frequency analysis (RFA), the implant stability quotient (ISQ) was measured to assess implant stability.

Methodology

This split-mouth, randomized, single-blinded clinical trial included 20 participants undergoing dental implant placement. The test group received LLLT while the control group had no laser treatment. Implant stability was assessed using RFA at one, two, four, and twelve weeks post-implant placement. Statistical analysis involved descriptive statistics, repeated-measures analysis of variance (ANOVA), and t-tests.

Results

The repeated-measures ANOVA analysis showed significant differences in the ISQ values between the LLLT group and the control group at two weeks and three months post-implant placement. The LLLT group exhibited higher ISQ values, indicating greater implant stability and improved osseointegration compared to the control group. These findings suggest the potential benefits of LLLT in enhancing dental implant outcomes.

Conclusions

LLLT shows promise in improving dental implant outcomes, with enhanced stability and osseointegration. Further research is needed to validate these results and integrate LLLT into routine dental implant procedures.

## Introduction

Dental implants have revolutionized the field of restorative dentistry by offering a reliable solution for replacing missing teeth. The application of photobiomodulation techniques using low-level laser therapy (LLLT) has gained momentum as researchers have now recognized the potential of light energy to promote osteoblast activity, thereby improving implant stability. Photobiomodulation through low-level lasers is mediated by the mitochondrial enzyme cytochrome C oxidase (CCO) and includes the absorption of near-infrared light to activate certain chromophores and trigger signaling pathways. This activation immediately increases ATP generation and promotes CCO dissociation from inhibitory nitric oxide, increasing ATP availability and activating activities such as vasodilation and angiogenesis for analgesic effects. It also promotes bone and connective tissue remodeling, possibly via increased mitochondrial reactive oxygen species and increased vascular activity, resulting in accelerated bone turnover and improvement in implant stability.

This biphasic response is consistent with the Arndt-Schulz rule, which states that various stimulus intensities might have distinct effects on vital activity. As previous studies [1-3] have showcased positive outcomes on implant stability with the use of LLLT, attention has now shifted toward optimizing the parameters and protocols for its application around dental implants to enhance implant stability [2,4,5].

Although multiple studies have reported success with LLLT, there is not one standardized protocol followed in these studies. Moreover, very limited evidence and randomized clinical trials are available evaluating the effectiveness of LLLT when used in the non-contact mode. The time of assessment is varied across the literature. A few studies have suggested a single-time application of LLLT whereas a few have suggested multiple.

More recently, LLLT has emerged as a particularly promising method within photobiomodulation, showcasing impressive effectiveness in improving dental implant outcomes [6,7]. The utilization of low-level lasers has gained popularity due to their unique ability to deeply penetrate tissues and induce beneficial effects [3,8]. By stimulating cellular metabolism, promoting angiogenesis, and modulating inflammatory responses, LLLT contributes to enhanced tissue healing and improved osseointegration of dental implants [9]. It achieves this through the emission of low-intensity lasers that produce coherent light at specific wavelengths, ensuring minimal thermal effects on the surrounding tissues. The remarkable potential of LLLT in promoting tissue repair and accelerating the healing process has sparked considerable interest among dental professionals and researchers alike [10,11]. Consequently, there is a growing desire to evaluate its effectiveness in enhancing dental implant outcomes, ultimately leading to improved patient satisfaction.

This study aimed to assess the effectiveness of photobiomodulation on implant stability using LLLT around dental implants at two, four, six, eight, ten, and twelve days. The null hypothesis was that there was no difference in the implant stability quotient (ISQ) recorded with and without LLLT.

## Materials and methods

Sample size and study design

**Figure 1:**
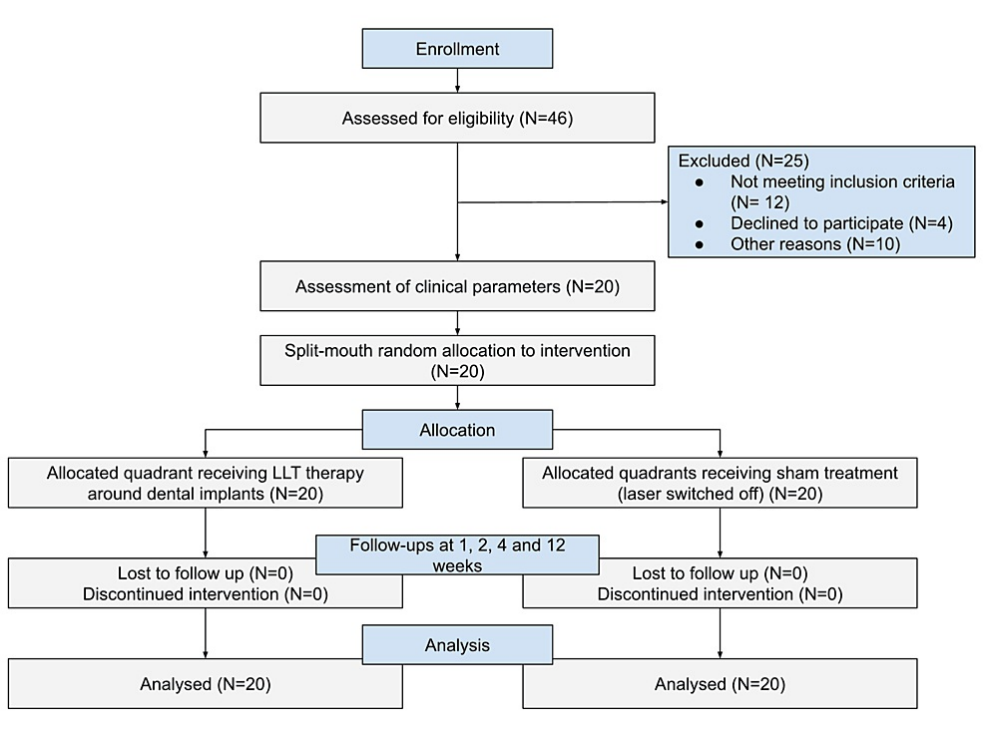
Flowchart depicting the enrolment, randomization, and patient allocation of each group.

This split-mouth, randomized clinical trial was conducted at Saveetha Dental College and Hospitals, Chennai, India, after receiving ethical approval from the Institutional Ethical Committee Review Board (approval number: IHEC/SDC/PROSTHO-2002/23/147) and under the Indian Clinical Trials Registry (CTRI/2023/10/058685). The sample size of 18 was estimated using G*Power 3.1.9.3 for Mac OS X® [12] using the data from a previous publication [13]. Assuming a normal distribution, the effect size (dz = 1.5004) and the sample size were estimated at an alpha of 0.05 and a power of 0.95 (1−b error probability). A total of 20 participants were selected to account for the loss of follow-up mid-treatment. Written consent forms were obtained from all participants before starting the procedures. A total of 20 participants remained at the end of the study. This study followed the CONSORT guidelines where 46 participants between the ages of 18 and 65 requiring replacement of one missing tooth on each side (right and left) were selected from the outpatient pool from March 23, 2022, to June 15, 2023, using simple random sampling based on the inclusion and exclusion criteria (Figure 1).

Inclusion and exclusion criteria

This inclusion criteria focused on selecting suitable participants aged between 18 and 65 years with bilateral edentulousness in opposing quadrants. Emphasis was placed on individuals in good health, devoid of any systemic conditions or periodontally compromised dentition. Another crucial factor was the presence of sufficient amounts of available alveolar bone, sufficient to accommodate the standard dental implant size (3.5 × 11.5 mm) for both implant sites. Exclusion criteria were established to identify individuals ineligible for participation. Patients with a history of systemic diseases or conditions, such as uncontrolled diabetes or immunodeficiency disorders, which could potentially compromise the success of dental implant surgery and osseointegration, were excluded. Furthermore, those who were smokers or users of tobacco products were not considered, given their propensity to hinder positive outcomes. Individuals with a history of radiation therapy to the head and neck region, pregnant or lactating mothers, and those who had previously received dental implants in the same region were also excluded. Lastly, individuals currently engaged in other clinical trials or studies that might interfere with the present study’s outcomes were ineligible, ensuring a focused and coherent participant group for accurate findings.

Randomization, allocation concealment, and blinding

Randomization was performed to assign the treatment order (experimental or control) to each patient’s dental implant placement sites (right and left sides) using a computer-generated randomization sequence (https://www.randomizer.org/#randomize) by an independent researcher not involved in the study. Allocation to either group was performed using a lottery method. Allocation concealment was done using opaque envelopes that were sealed until the time of surgical procedure. Blinding the patients and surgeons performing surgery was not possible. Outcome evaluation was done by another researcher (DG), who was blinded to the allocation, making it a single-blinded trial.

Clinical procedure

**Figure 2:**
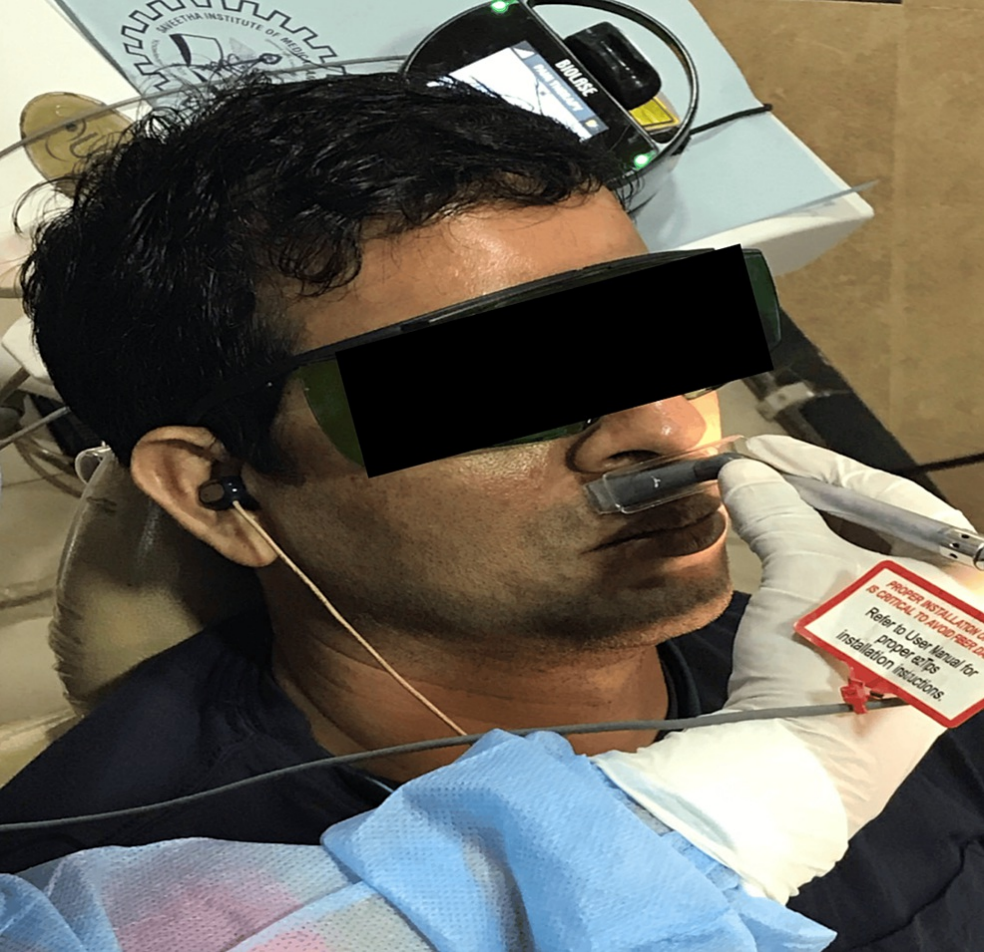
Laser application.

The surgical procedure was performed by a dental surgeon specialized in oral implantology with the assistance of dental students specialized in one of the three departments in the field using a standardized protocol as per the institutional guidelines. Local anesthesia was administered, after which an incision was made in the region of the median ridge and fissures. This was followed by a full-thickness flap elevation with direct access to the bone tissue and subsequent sequential drilling with an Osstem drill. Implants measuring 3.511.5 mm (Osstem implant TS III SA, Osstem Implant Co., Seoul, Korea) were placed and broken sutures were given. After the implants were placed, the implant sites were randomly divided into the following two groups: the experimental group, where the dental implant placement sites received photobiomodulation using Biolase laser therapy; and the control group, where the dental implant placement sites did not receive any additional intervention and served as the control for comparison. It is important to note that the test and control implants were not placed side by side in any patient to avoid possible interference between groups. In the test group, a diode laser (epic10, Biolase, Inc., Irvine, CA, USA) was used for irradiation of the soft tissue around the implants in a separate room. Protective glasses were worn by both the patient and the clinician. A 6 mm spot size deep tissue handpiece was used, with laser parameters set at a 940 nm wavelength, 100 mW output power, and a power density of 354.6 mW/cm^2^ in continuous wave mode. The laser irradiation was done for 40 seconds on each side of the implant (buccal and palatal) at an energy density of 14.18 J/cm^2^ per side, totaling 8 J per session. The laser treatment was repeated on the second, fourth, sixth, eighth, tenth, and twelfth days postoperatively, resulting in a cumulative dose of 56 J, to aid in initial healing and promote osteoblastic proliferation. The same procedure was followed with the laser off in the second group, and protective glasses were worn by both the patient and the clinician throughout (Figure 2).

**Figure 3:**
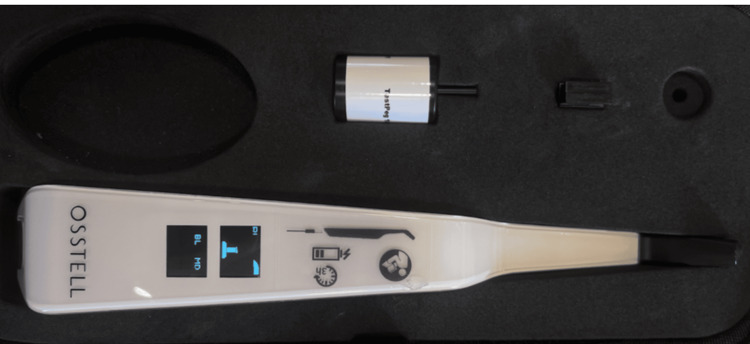
Osstell Beacon device used for recording the implant stability quotient values.

This procedure was done postoperatively. After the surgical procedure, the healing caps were taken off in each group to evaluate the primary stability of the implants. The smart peg from the resonance frequency analysis (RFA) device (Osstell Beacon, Gross Mendelsohn & Associates, Baltimore, MD 21230) was inserted into the implant fixture (Figure 3).

The transducer’s head was positioned vertically on the smart peg to determine the ISQ. Assessment of stability was done at baseline and one, two, four, and twelve weeks after placement, as each of these intervals signified an important phase of osseointegration with initial cellular proliferation, vascular changes, soft tissue healing, and bone healing.

Outcome

The ISQ values were measured at one, two, four, and twelve weeks post-implant placement using RFA (Osstell system).

Statistical analysis

Data were collected and tabulated in Google Sheets. Data monitoring and quality control procedures were implemented to ensure accuracy. The collected information was analyzed using SPSS version 26.0 (IBM Corp., Armonk, NY, USA) via descriptive statistics. All the data obtained were tested for normality using the Kolmogorov-Smirnov test. Repeated-measures analysis of variance (ANOVA) was done to evaluate changes in implant stability over different time intervals, and unpaired t-tests were used to compare the two groups at the end of 12 weeks.

## Results

**Figure 4:**
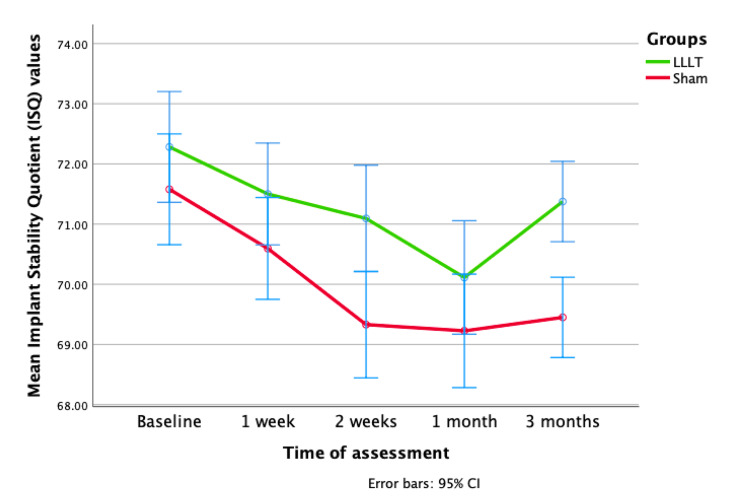
Baseline mean ISQ values were 72.28 for LLLT and 71.58 for sham. At one, two, and four weeks, LLLT showed decreasing trends (71.50, 71.09, and 70.11) compared to sham (70.60, 69.33, and 69.23). At 12 weeks, LLLT rebounded to 71.38, while sham remained at 69.45. ISQ = implant stability quotient; LLLT = low-level laser therapy

All 20 participants completed the study with no dropouts. None of the patients reported any adverse effects during or after treatment. The primary outcome evaluated was the improvement in the ISQ over 12 weeks. The repeated-measures ANOVA indicated no disparity in the recorded ISQ during the initial week. Nevertheless, a significant difference (p < 0.05) between the two groups emerged distinctly at the two-week and three-month marks, indicating an improvement in the ISQ during that period. The findings underscore variations of significance in the ISQ values over the analyzed time intervals, displaying prominent linear and quadratic trends (Figure 4). Furthermore, substantial variations (p < 0.05) in the average ISQ values between the LLLT and sham groups were observed, substantiated by data presented in Table 1.

**Table 1:** Pairwise comparison of changes in the observed ISQ values at given observation periods. P-value derived using the paired t-test. ISQ = implant stability quotient

Groups	Mean difference	Standard error	95% CI		P-value
			Lower	Upper	
Baseline vs. 1 week	0.882	0.312	0.250	1.514	0.007*
Baseline vs. 2 weeks	1.718	0.421	0.865	2.571	0.000*
Baseline vs. 1 month	2.260	0.458	1.333	3.188	0.000*
Baseline vs. 3 months	1.517	0.423	0.661	2.373	0.001*
1 week vs. 2 weeks	0.837	0.238	0.355	1.318	0.001*
1 week vs. 1 month	1.379	0.428	0.513	2.244	0.002*
1 week vs. 3 months	0.635	0.391	-0.157	1.428	0.113
2 weeks vs. 1 month	0.542	0.398	-0.264	1.348	0.181
2 weeks vs. 3 months	-0.200	0.391	-0.157	1.428	0.113
1 month vs. 3 months	-0.743	0.339	-0.886	0.485	0.557

## Discussion

This split-mouth, randomized, single-blinded clinical trial highlights the effectiveness of LLLT in improving dental implant outcomes. The decision to evaluate implant stability at specific time intervals (one, two, four, and twelve weeks after implant placement) is grounded in the typical healing and osseointegration process observed in dental implants [14]. These time points coincide with essential stages of tissue healing, bone formation, and implant integration. Assessing implant stability during these intervals offers valuable insights into the progress of osseointegration and the increasing implant stability as it integrates with the surrounding bone [15]. The findings of this study revealed that the LLLT group exhibited superior implant stability compared to the control group. Thus, the null hypothesis was rejected. These positive outcomes further support the potential benefits of utilizing LLLT as an adjunct therapy in dental implantology to enhance implant success rates and accelerate the healing process.

This study focused on assessing outcomes at specific and clinically relevant intervals, indicating it is important to keep in mind that previous research on photobiomodulation in dental implantology has also yielded promising results; however, those studies either used different surface-treated implant systems or assessed outcomes at different or less relevant intervals [16-18]. It is also worth noting that a few studies [13,19-21] have shown conflicting or negative results, indicating that the effectiveness of LLLT may vary based on individual patient factors and other variables. Despite this, this study contributes to the prevailing consensus among the majority of studies, leaning toward LLLT being beneficial in enhancing dental implant stability outcomes.

LLLT has been investigated for its potential to stimulate osteoblast activity. Osteoblasts are specialized cells responsible for bone formation and mineralization. LLLT involves the application of low-power laser or light-emitting diodes to targeted tissues, and it has been suggested that this form of light energy can modulate cellular functions. Studies [22-24] propose that LLLT may influence osteoblasts at a cellular level by enhancing mitochondrial function, increasing adenosine triphosphate production, and promoting the release of reactive oxygen species. These cellular responses, in turn, may activate various signaling pathways, including those involving growth factors and transcription factors, ultimately leading to increased osteoblast proliferation and differentiation. While the precise mechanisms underlying LLLT’s effects on osteoblasts are still a subject of ongoing research [25], the current understanding suggests a complex interplay of cellular events that contribute to the observed stimulation of bone-forming activities.

While this study adhered to the recommended guidelines, some limitations should be considered, such as the small sample size. Due to the nature of the intervention, individual patient factors, such as variations in bone quality, healing capacity, and systemic health, might have impacted the outcomes, emphasizing the importance of controlling for these variables in future research to gain more comprehensive insights [26]. The positive outcomes and growing evidence supporting LLLT’s effectiveness suggest promising implications for dental implantology. However, further research and larger trials are needed to validate these findings. Exploring LLLT’s long-term effects and its application in specific patient populations with compromised bone quality or systemic conditions can refine protocols and optimize outcomes.

## Conclusions

Photobiomodulation using LLLT may positively influence tissue healing and improve ISQ, ultimately accelerating osseointegration of the implant to the bone. By understanding the potential benefits and limitations of this emerging therapy, practitioners can optimize photobiomodulation protocols and ultimately enhance implant success rates and patient outcomes and satisfaction.
